# Leap Motion Controller Video Game-Based Therapy for Upper Extremity Motor Recovery in Patients with Central Nervous System Diseases. A Systematic Review with Meta-Analysis

**DOI:** 10.3390/s21062065

**Published:** 2021-03-15

**Authors:** Irene Cortés-Pérez, Noelia Zagalaz-Anula, Desirée Montoro-Cárdenas, Rafael Lomas-Vega, Esteban Obrero-Gaitán, María Catalina Osuna-Pérez

**Affiliations:** 1 Centro Médico “Avenida II”, C/Julio Burell 18, 23700 Linares, Spain; icp00011@red.ujaen.es; 2 Department of Health Sciences, University of Jaén, Paraje Las Lagunillas s/n, 23071 Jaén, Spain; nzagalaz@ujaen.es (N.Z.-A.); dmc00047@red.ujaen.es (D.M.-C.); rlomas@ujaen.es (R.L.-V.); mcosuna@ujaen.es (M.C.O.-P.)

**Keywords:** leap motion controller, virtual reality, central nervous system diseases, upper extremity, motor function, grip strength, gross motor dexterity, fine motor dexterity, meta-analysis

## Abstract

Leap Motion Controller (LMC) is a virtual reality device that can be used in the rehabilitation of central nervous system disease (CNSD) motor impairments. This review aimed to evaluate the effect of video game-based therapy with LMC on the recovery of upper extremity (UE) motor function in patients with CNSD. A systematic review with meta-analysis was performed in PubMed Medline, Web of Science, Scopus, CINAHL, and PEDro. We included five randomized controlled trials (RCTs) of patients with CNSD in which LMC was used as experimental therapy compared to conventional therapy (CT) to restore UE motor function. Pooled effects were estimated with Cohen’s standardized mean difference (SMD) and its 95% confidence interval (95% CI). At first, in patients with stroke, LMC showed low-quality evidence of a large effect on UE mobility (SMD = 0.96; 95% CI = 0.47, 1.45). In combination with CT, LMC showed very low-quality evidence of a large effect on UE mobility (SMD = 1.34; 95% CI = 0.49, 2.19) and the UE mobility-oriented task (SMD = 1.26; 95% CI = 0.42, 2.10). Second, in patients with non-acute CNSD (cerebral palsy, multiple sclerosis, and Parkinson’s disease), LMC showed low-quality evidence of a medium effect on grip strength (GS) (SMD = 0.47; 95% CI = 0.03, 0.90) and on gross motor dexterity (GMD) (SMD = 0.73; 95% CI = 0.28, 1.17) in the most affected UE. In combination with CT, LMC showed very low-quality evidence of a high effect in the most affected UE on GMD (SMD = 0.80; 95% CI = 0.06, 1.15) and fine motor dexterity (FMD) (SMD = 0.82; 95% CI = 0.07, 1.57). In stroke, LMC improved UE mobility and UE mobility-oriented tasks, and in non-acute CNSD, LMC improved the GS and GMD of the most affected UE and FMD when it was used with CT.

## 1. Introduction

Central nervous system diseases (CNSDs) include a wide group of diseases that affect the brain (cerebral hemispheres, diencephalon, brain stem, and cerebellum) or the spinal cord, causing motor, balance, and cognitive impairments [1]. CNSD can be due to different causes, including vascular damage to brain areas, such as ischemic or hemorrhagic stroke [2], developmental and non-progressive neurological disorders, such as cerebral palsy [3], or neurodegenerative causes, such as multiple sclerosis, Alzheimer’s disease, and Parkinson’s disease [4]. All of the CNSDs mentioned above share disabling symptoms such as difficulties in voluntary extremity movement [5], gait and balance disorders [6], and decreased functional capacity and personal autonomy [7]. The most common disabling alterations in CNSDs are motor impairments in the upper extremities (UE) that reduce the range of motion (ROM) [8] and produce muscle weakness and/or spasticity [9]. In addition, reductions in grip strength (GS) [10], manual skills [8], gross and fine motor dexterity (GMD and FMD) [11], and tactile discrimination [12] alter the ability to perform activities of daily living (ADL), such as dressing, eating, or writing [13].

Currently, conventional therapy (CT) is the most commonly used approach to improve the UE motor impairments caused by CNSD [14]. Specifically, physiotherapy and occupational therapy are the most commonly used CTs in neurorehabilitation for stroke and other CNSDs [15]. CT is based on the practice of passive (at first, when the patient is most impaired) and active, high-intensity and repetitive tasks conducted by a therapist with the aim of activating damaged brain areas to promote neural plasticity [16]. Scientific literature suggests that the effect of CT may be limited (in long-term therapy, patients sometimes show difficulties adhering to treatment due to lack of motivation [17]). This technology can help to resolve these difficulties with novel therapeutic approaches [18]. In recent years, technological development has enabled the inclusion of new technologies in neurorehabilitation. Virtual rehabilitation using virtual reality (VR) devices [19] has emerged as a novel promising modality for motor rehabilitation in subjects with CNSD [20]. VR technology allows the patient to be integrated into a virtual environment that closely resembles the real environment through a computer and interact with it [21]. Non-immersive VR allows patients to experience a virtual environment as observers [22] and to interact with the virtual environment presented on the computer screen through the use of the mouse, keyboard, or other haptic devices that allow interaction with the game [23] in a low-cost experience [24]. Non-immersive VR devices are among the most promising VR tools for designing physiotherapy programs due to the great potential shown for training UE motor function [25]. Different studies have assessed the effect of the clinical application of non-immersive VR in patients who have suffered a CNSD [26]. Although stroke is the leading CNSD in which non-immersive VR has been used [27], in other CNSDs that cause motor impairments such as cerebral palsy [28], multiple sclerosis [29], Parkinson’s disease [30] or spinal cord injury [31], non-immersive VR has been extensively studied with promising results. However, to train the disabled manual skills more specifically (e.g., GS, GMD and FMD), it is necessary to use VR haptic devices, such as the Leap Motion Controller (LMC) [32].

The LMC is a consumer-grade and contact-free interaction [33] developed by Leap Motion (Leap Motion Inc., San Francisco, CA, USA [34], https://leapmotion.com, accessed on 1 February 2021) that does not require sensors to be placed on the participant’s body [35]. The LMC was designed to detect, recognize, and capture hand gestures and finger positions in interactive software applications [36]. In addition, the LMC allows the tracking of the arm, wrist, and hand positions of up to four participants [36]. This device incorporates three infrared sensors and two charge-coupled device cameras for computing hand geometry measurements for person-related hand recognition [37]. The LMC does not emit any structured light or create a depth scene map unless the LMC obtains the hand and finger positions from the stereo-vision images, and all mathematical calculations are carried out on the host computer using a proprietary algorithm [36]. The sensor accuracy in fingertip position detection is approximately 0.01 mm [36]. Fingertip positions over the LMC are measured in Cartesian coordinates relative to the center of the LMC in a right-handed coordinate system. The LMC is equipped with a high-precision optical tracking module that allows a hand tracking speed up to 200 frames per second in a 150° field of view with approximately eight cubic feet of interactive 3D space, allowing the perfect integration of one or both hands into the field [38]. The LMC generates a virtual representation of the UE on the computer screen and indicates to the patient what task should be performed [35]. Compared to other motion capture systems, such as Kinect^®^ (Microsoft Corp., Redmond, WA, USA), which is the most widely used body recognition device in balance and gait analysis [39], LMC shows several advantages, including its low cost [34], its small size, its ease of use and installation [40], and the wide variety of engineering applications that can be used, such as physical rehabilitation and assessment [41,42] and medical education [43]. Several studies have assessed the accuracy of manual motion tracking using LMC [44,45]. Smeragliuolo et al. [46] reported that the LMC is accurate for wrist flexion/extension and radial and cubital deviation, although it is less precise for arm supination and pronation. Chophuk et al. [47] suggested small error angles in fingers using LMC to recognize real finger movement. Recently, Fonk et al. [48] reported that the LMC is able to provide a correct estimation of the orientation of the hand bones and joint positions to be reproduced with precision in software with biomechanical applications.

To date, different studies have analyzed the validity [49], feasibility [50], and usability [32] of LMC for use in neurorehabilitation. Several RCTs have assessed the effect of immersive or non-immersive VR on UE motor function recovery [51,52,53], and consequently, some reviews have been carried out [54,55,56]. However, the use of LMC as a VR tool in UE neurorehabilitation has been less studied [57,58]. Therefore, the objective of the present review was to retrieve published evidence to analyze the effect of video game-based therapy using LMC to improve UE motor function in patients with acute and non-acute CNSD. Second, we assessed the effect of LMC on UE motor function when it was used alone or in combination with CT.

## 2. Materials and Methods

### 2.1. Protocol Review

A systematic review with meta-analysis was performed following the preferred reporting items for systematic reviews and meta-analyses (PRISMA) statement proposed by Moher et al. 2009 [59] and the Cochrane Handbook for Systematic Reviews of Interventions of Higgins and Green [60]. In addition, the protocol of this review has been registered in PROSPERO (International Prospective Register of Systematic Reviews) with the registration number CRD42020200771 [61].

### 2.2. Search Strategy and Data Sources

A bibliographic search was conducted in PubMed Medline, Web of Science (WOS), Scopus, CINAHL Complete, and PEDRO (Physiotherapy Evidence Database) independently by two authors (I.C.-P. and D.M.-C.) between November 2020 and January 2021. Furthermore, the authors also performed a search in the reference lists of the full-text articles retrieved with the original search strategy, in the gray literature and in expert documents. The search strategy was based on the use of the PICOS tool proposed by the Cochrane Library [60]: (1) population, subjects with CNSD; (2) intervention, video game-based therapy using LMC; (3) comparison, CT or no intervention; (4) outcomes, UE motor function (mobility, GS, GMD, and FMD); and (5) study design, randomized controlled trials. The main terms included in our search strategy were provided by the Medical Subjects Headings (MeSH), the EBSCOhost thesaurus, and entry terms, such as “leap motion controller” and “upper extremity”. These terms were combined using Boolean operators (AND/OR) following the search specifications of each database used. To perform this search, the authors did not use filters related to the publication date and language. Any lack of consensus related to the search was solved by the consultation with a third author with expertise in the bibliographical search (E.O.-G.). Duplicated studies retrieved were excluded for the next step (study selection). Table 1 shows the bibliographic strategy search used in each database.

### 2.3. Study Selection and Inclusion Criteria

The study selection process was carried out by two authors (I.C.-P. and D.M.-C.). Each of these authors independently screened each article retrieved by title/abstract. When a reference was selected as a potentially eligible study by one of the reviewers, it was examined in detail. Disagreements arising during this phase were resolved by consulting with a third author (M.C.O.-P).

The inclusion criteria proposed were as follows: (1) randomized controlled trials (RCTs); (2) RCTs with a sample composed of patients diagnosed with a CNSD; (3) studies that used LMC video game-based therapy as an intervention (alone or combined with other therapy); (4) studies in which the comparison group did not receive an intervention or received a therapy different from LMC; (5) studies that assessed different outcomes related to the motor function of the UE; and (6) studies that provided data to perform the meta-analysis. As exclusion criteria, we used (1) RCTs with a population study that included CNSD and other pathologies and (2) studies with only one group.

### 2.4. Data Extraction

The data extracted from the included studies were collected using a Microsoft Excel standardized collection form by two authors (N.Z.-A. and I.C.-P.). A third author (R.L.-V.) resolved any possible discrepancies related to the extracted data.

From each study, we extracted the following data: (1) data related to the general characteristics of each study (authorship and publication date, country, study design, total sample size, type of CNSD, evolution, and number of groups included); (2) characteristics of each group (number of participants, age and sex); (3) characteristics of the intervention in experimental and comparison groups (type of intervention, number of weeks, days/week and minutes of each session); and data regarding outcomes (type of outcome, test employed for evaluation, time between the end of the therapy and the last assessment and quantitative data for the meta-analysis). Data used in our meta-analysis were the means and their standard deviations and/or mean differences between groups and *p*-values. When a study did not provide data related to standard deviation, these data could be obtained from other data presented in the study, such as range, interquartile range or standard error, according to standardized statistical procedures [60,62].

### 2.5. Outcomes

The main outcome assessed was UE motor function (in terms of mobility, GS, or hand dexterity). Specifically, different outcomes related to UE motor function were susceptible to assessment, such as UE mobility, the UE mobility-oriented task, the GS, and the GMD and FMD.

### 2.6. Assessment of Evidence Quality and Risk of Bias

In this study, the authors assessed the quality evidence of our findings and the risk of bias. First, the Grading of Recommendations Assessment, Development, and Evaluation (GRADE) system [63] was used to analyze the overall quality of the evidence in each meta-analysis. This scale assesses different aspects, such as the risk of bias of each study, inconsistency, indirectness, imprecision, and publication bias risk. According to the suggestions of Meader et al. [64] in the GRADE checklist, all these items were assessed, except risk of bias, which was analyzed using the Cochrane Collaboration Risk of Bias Tool [65]. The assessment of the risk of publication bias is detailed in the statistical analysis section. Inconsistency was assessed through the level of heterogeneity [66] (see the Statistical Analysis section). To assess the precision, we took into account the number of participants per study (low, <100 participants; medium, 300–100 participants; and high, >300 participants) and the number of included studies (large, >10 studies; moderate, 10–5 studies; and small, <5 studies) [63].

The Cochrane Collaboration Risk of Bias Tool was chosen to analyze the risk of bias of the individual studies included in the present review [60]. This scale is formed by seven items (selection, performance, detection, attrition, reporting, and other bias) and classifies the risk of bias as low, uncertain (when studies did not provide detailed information or description), and high [65].

Two reviewers (D.M.-C. and E.O.-G.) independently assessed the methodological quality. The quality evidence of each meta-analysis was downgraded one level according to the limitations found. However, if several limitations were present in the findings, the quality evidence was downgraded two levels. Finally, we established a meta-analysis classified into four levels of evidence: (1) high when the findings are robust due to the absence of limitations; (2) moderate if limitations may change the generalization of our results; (3) low if the presence of various limitations decreases the level of confidence in our results; and (4) very low when the estimation of the effect is very uncertain. Any lack of consensus regarding downgrading the level of evidence was resolved by a third author (M.C.O.-P.).

### 2.7. Statistical Analysis

The meta-analyses were performed by two authors (E.O.-G. and I.C.-P.) using Comprehensive Meta-Analysis 3.3.070 (Biostat, Englewood, NJ, USA) [67]. We followed the recommendations of Cooper et al. in The Handbook of Research Synthesis and Meta-Analysis [68]. To reduce the impact heterogeneity in each study, we used the random-effects model described by DerSimonian and Laird [69] to estimate the effect of the intervention and its 95% confidence interval (95% CI) with the aim of improving the generalization of our findings. Cohen’s standardized mean difference (SMD) was used to calculate the pooled effect [70], which can be interpreted as small (SMD = 0.2), moderate (SMD = 0.5), and large (SMD > 0.8) [71] and can be displayed as a forest plot [72]. The risk of publication bias was assessed with the symmetry or asymmetry present in the funnel plot [73], with Egger’s test (where *p* < 0.1 suggests risk of publication bias) [74] and with the Trim and Fill method [74]. Related to publication bias, the quality level of evidence was not downgraded if the adjusted pooled effect, according to the Trim and Fill method, varied less than 10% with respect to the original and raw pooled effect, although the funnel plot was slightly asymmetric. Heterogeneity was analyzed with the Cochrane-Q test; the degree of inconsistency (*I^2^*) may be small (<25%), medium (25–50%), or large (>50%), and *p* < 0.1 indicates the presence of heterogeneity [66,75].

### 2.8. Additional Analysis

A sensitivity analysis was performed using the leave-one-out method with the aim of assessing the contribution of each study to the overall pooled effect in each analysis [68]. In addition, subgroup analysis was carried out to assess the effect of LMC when it was used alone or combined with CT.

## 3. Results

### 3.1. Study Selection

**Figure 1:**
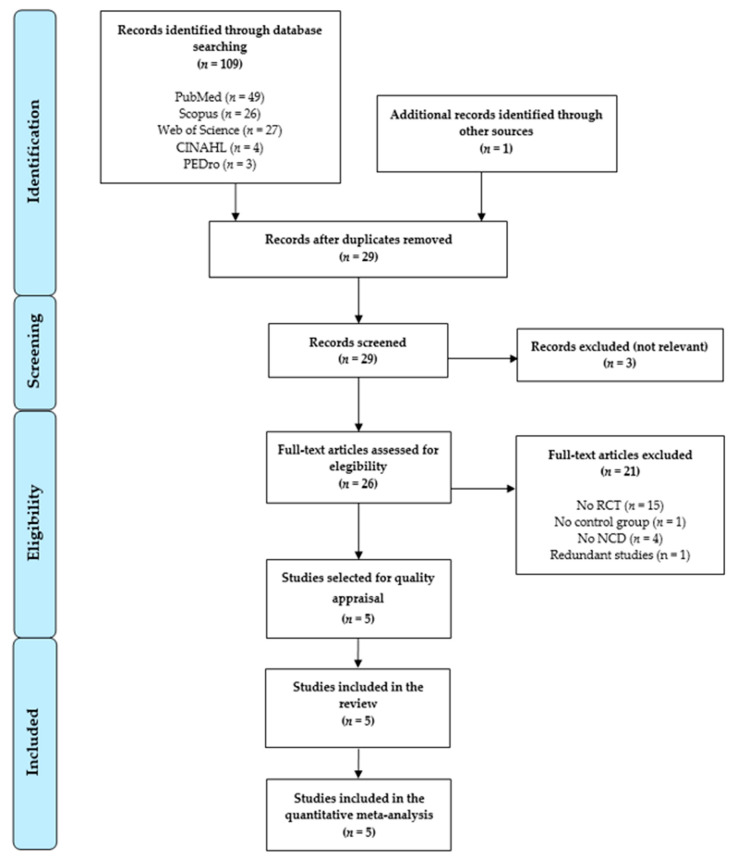
Preferred reporting items for systematic reviews and meta-analysis (PRISMA) flow chart.

A PRISMA flow chart (Figure 1) was constructed to display the results of the bibliographic search and the study selection phases. A total of 109 studies were retrieved from databases, and 1 study was retrieved from the gray literature. After duplicates were removed (*n* = 81), 29 studies were screened by title/abstract. Three studies were excluded by title/abstract. Of the 26 remaining studies, 21 were excluded for not meeting the inclusion criteria (reasons in Figure 1). Ultimately, 5 studies were included in the quantitative synthesis of this review [76,77,78,79,80].

### 3.2. Main Characteristics of the Studies Included in the Review

Five studies with 22 independent comparisons provided data from 174 participants (mean age of 48.35 ± 21.45 years old), of whom 91 participants (57.48 ± 3.76 years old) were diagnosed with stroke [79,80], 30 with cerebral palsy (11 ± 0.09 years old) [76], 30 with multiple sclerosis (46.26 ± 5.09 years old) [77], and 23 with Parkinson’s disease (69.7 ± 5.55 years old) [78]. The studies were undertaken in different countries: Turkey (2 studies) [76,80], Spain (2 studies) [77,78], and China (1 study) [79]. Eighty-nine patients (48.66 ± 19.67 years old) received an experimental therapy that included video game-based LMC, which was used alone [78,80] or combined with other therapies [76,77,79]. Cuesta-Gómez et al. [77] and Fernández-González et al. [78] established a rehabilitation program with six LMC-based videogames (“Piano Game”, “Reach Game”, “Sequence Game”, “Grasp Game”, “Pinch Game”, and “Flip Game”) in different virtual environments using LMC to capture and train the UE movement and skills both unilaterally and bilaterally. Wang et al. [79] established an experimental protocol using six LMC-based video games, such as “Piano Game”, “Petal-picking game”, “Robot-assembling game”, “Object-catching”, “Firefly game” and “Bee-batting game”. Avcil et al. [76] used 2 Nintendo^®^ Wii games (tennis and boxing games) and 2 LMC-based videogames (“CatchAPet” and “Leapball”) to try to improve GS and hand function. Finally, Ögun et al. [80] employed four LMC-based video games (cube handling, decorating a tree with leaves, kitchen experience, and drumming) focused on hand grip and manipulative UE movements. The comparison group was composed of 75 subjects (48.10 ± 21.05 years old) who received only CT (physiotherapy or occupational therapy) [76,77,78,79] or CT with more passive image visualization [80] as a control intervention. The durations of the interventions based on LMC therapy were 4 [79], 6 [78,80], 8 [76], and 10 weeks [77]. The numbers of sessions per week were 2 in 2 studies [77,78], 3 in 2 other studies [76,80], and 5 in one study [79]. Finally, 3 studies performed daily sessions of 60 min [76,77,80], 1 study performed daily sessions of 45 min [79], and another study performed daily sessions of 30 min [78]. All studies included in this review were RCTs and assessed the different aspects of UE motor function, such as UE mobility and UE mobility-oriented task using the Fugl–Meyer Assessment of the Upper Extremity, the Action Research Arm Test, and the Wolf Motor Function Test [79,80]; the GS [76,77,78] using a dynamometer; GMD [76,77,78] with the Minnesota Manual Dexterity Test and the Box and Block Test; and finally the FMD [76,77,78] using the Duruoz Hand Index and the Purdue Pegboard Test. The main characteristics of the studies included in this review are summarized in Table 2.

### 3.3. Risk of Bias Assessment

The assessment of the risk of bias and methodological quality of the studies included in the present review is shown in Table 3. Two studies were susceptible to showing a higher risk of bias [76,78]. No study was able to blind the type of therapy to the participants in either group [76,77,78,79,80]. The risk of detection bias was increased in one study [78] and probably in another [76]. All studies showed a risk of selection and performance bias [76,77,78,79,80]. In general, the overall quality of the studies included could be moderate due to the possible risk of selection, performance, and detection bias.

### 3.4. Effect of LMC-Video Game Based Therapy on the Recovery of UE Mobility in Patients with Stroke

**Figure 2:**
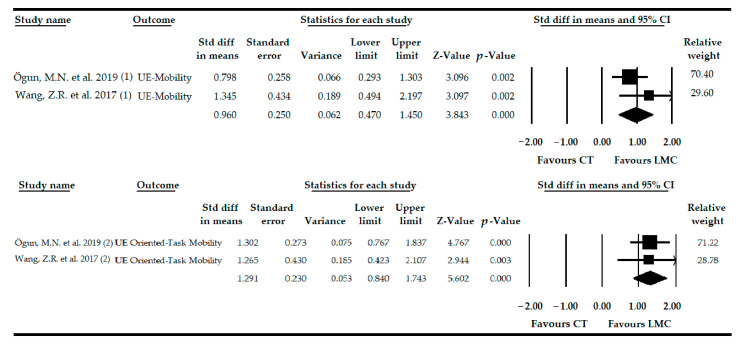
Forest Plot of the effect of Leap Motion Controller (LMC)-based therapy on recovery of the upper extremity (UE) mobility and the UE mobility-oriented task in patients with stroke.

Two studies [79,80] with 2 independent comparisons provided data from 91 patients diagnosed with stroke. Forty-six patients (58.39 ± 4.36 years old) received LMC therapy in the intervention group, and 45 participants (56.57 ± 4.49 years old) received other therapies as a control intervention. Very low-quality evidence of a large effect of LMC-based therapy (SMD = 0.96; 95% CI = 0.47, 1.45; *p* < 0.001) was shown on UE mobility in patients with stroke (Table 4, Figure 2). Although the risk of publication bias was not assessed, it must be considered high due to the low number of studies included. Heterogeneity was not present (*I^2^* = 0%, *p* = 0.31), and the precision of the findings was low (45.5 participants per study). The leave-one-out method yielded pooled estimates that varied 37% when compared to the original pooled effect.

After a subgroup analysis, according to the specific therapy used, our findings showed very low-quality evidence of a higher effect size of the combined use of LMC than when LMC was used alone (SMD = 1.34; 95% CI = 0.49, 2.19; *p* = 0.002) (SMD = 0.79; 95% CI = 0.29, 1.30; *p* = 0.002), both versus CT.

### 3.5. Effect of LMC-Video Game Based Therapy to Restore the UE Mobility-Oriented Task in Patients with Stroke

Two studies [79,80] provided 2 independent comparisons with data from 91 patients diagnosed with stroke. LMC was included as an experimental intervention in the intervention group composed of 45 participants (56.57 ± 4.49 years old). Our findings showed very low-quality evidence of a large effect (SMD = 1.29; 95% CI = 0.84, 1.74; *p* < 0.001) of the use of LMC therapy on UE mobility-oriented task in subjects with stroke (Table 4, Figure 2). Publication bias was not assessed due to the low number of studies included. Heterogeneity was not present (*I^2^* = 0%, *p* = 0.94), and the precision level was low (45.5 participants per study). After applying the leave-one-out analysis, the pooled effect varied by only 2% with respect to the original SMD.

After performing the subgroup analysis according to the specific use of LMC, our findings showed very low-quality evidence of a higher effect of LMC when it was combined with CT (SMD = 1.26; 95% CI = 0.42, 2.10; *p* = 0.003) or isolated (SMD = 1.30; 95% CI = 0.76, 1.83; *p* < 0.001) versus CT.

### 3.6. Effect of LMC-Video Game Based Therapy on Grip Strength in Non-Acute CNSDs

**Figure 3:**
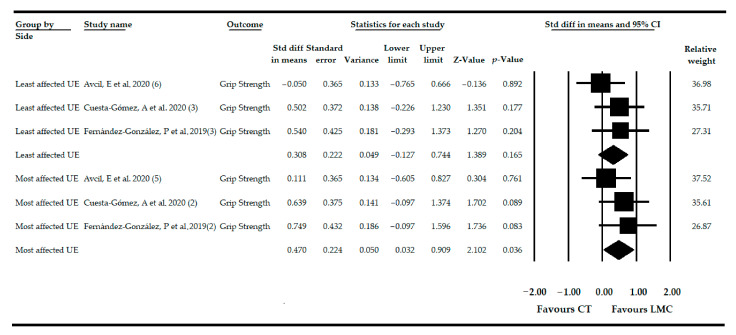
Forest Plot of the effect of LMC-based therapy on recovery of grip strength in patients with non-acute central nervous system disease (CNSD).

Three studies [76,77,78] provided data from 83 patients (42.32 ± 26.64 years old) with various non-acute CNSDs, where the effect of LMC was assessed to restore the GS in the most and least affected UE independently (3 independent comparisons on each UE side). Forty-three participants (42.18 ± 18.21 years old) received LMC as the experimental intervention, and 40 subjects (42.45 ± 31.18 years old) received CT as the comparison intervention. Our results suggested low-quality evidence of a medium-high effect of LMC (SMD = 0.47; 95% CI = 0.03, 0.90; *p* = 0.036) on the recovery of GS in the most affected UE compared to CT (Table 4, Figure 3). However, in the least affected UE, LMC did not show a statistically significant effect with low-quality evidence (SMD = 0.30; 95% CI = −0.12, 0.74; *p* = 0.165) compared to CT (Table 4, Figure 3). The risk of publication bias was not present in the most affected UE (Figure S1 in online supplementary files) and in the least affected UE (Figure S2 in online supplementary files). Heterogeneity was not present in any UE, and the precision level was low (27.6 participants per study). After the sensitivity analysis, the pooled effect varied by 45% with respect to the original SMD in the most affected UE and 67% in the least affected UE.

When the subgroup analysis was performed in the most affected UE, LMC did not produce an effect on GS independently if it was used alone (SMD = 0.38; 95% CI = −0.23, 1.00; *p* = 0.21) or combined with CT (SMD = 0.63; 95% CI = −0.09, 1.37; *p* = 0.089) compared to CT. Similar results were found in the least affected UE when LMC was used alone (SMD = 0.20; 95% CI = −0.36, 0.77; *p* = 0.482) or when a CT program was added (SMD = 0.50; 95% CI = −0.22, 1.23; *p* = 0.177).

### 3.7. Effect of LMC-Video Game Based Therapy on Gross Motor Dexterity in Non-Acute CNSD

**Figure 4:**
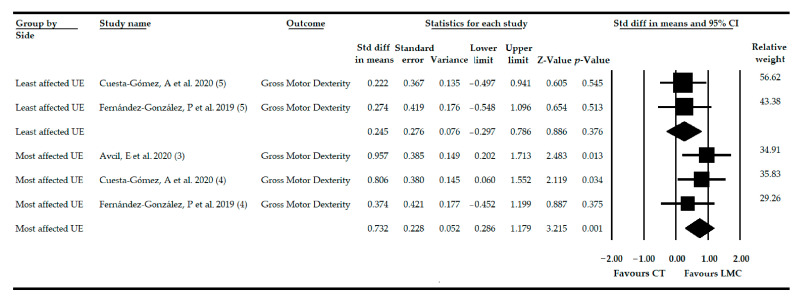
Forest Plot of the effect of LMC-based therapy on recovery of gross motor dexterity in patients with non-acute CNSD.

Three studies [76,77,78] reported data from 83 patients (42.32 ± 26.64 years old) with various non-acute CNSDs. The effect of LMC on GMD in the most affected UE was determined from data from 3 independent comparisons [76,77,78] and in the least affected UE from 2 comparisons [77,78]. Our findings suggested low-quality evidence of a medium-high effect of the use of LMC (SMD = 0.73; 95% CI = 0.28, 1.17; *p* = 0.001) on GMD in the most affected UE compared to CT (Table 4, Figure 4). However, when the meta-analysis was performed, taking into account the least affected UE, no statistically significant differences were found between the use of LMC and CT (SMD = 0.24; 95% CI = −0.29, 0.78; *p* = 0.376) (Table 4, Figure 4). The risk of publication bias was not present in the most affected UE (Figure S3 in online supplementary files ), and in the least affected UE, it could not be calculated. Heterogeneity was not present, and the precision level was low in both. Finally, the one study removed provided variations of 20% with respect to the original SMD in the most affected UE and 11% in the least affected UE.

After performing the subgroup analysis in the most affected UE, very low-quality evidence of a high effect of LMC and more CT (SMD = 0.80; 95% CI = 0.06, 1.55; *p* = 0.039) and a moderate effect of LMC used alone (SMD = 0.69; 95% CI = 0.12, 1.26; *p* = 0.018) were found in the comparison CT. However, when the subgroup analysis was performed in the least affected UE, LMC did not show an effect versus CT when it was combined with CT (SMD = 0.22; 95% CI = −0.49, 0.94; *p* = 0.671) or when it was used independently (SMD = 0.27; 95% CI = −0.54, 0.65; *p* = 0.625).

### 3.8. Effect of LMC-Video Game Based Therapy on Fine Motor Dexterity in Non-Acute CNSD

**Figure 5:**
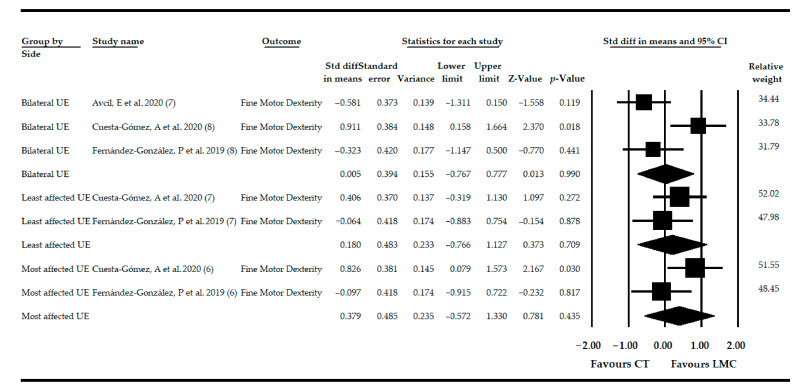
Forest Plot of the effect of LMC-based therapy on recovery of fine motor dexterity in patients with non-acute CNSD.

Three studies [76,77,78] provided data from 83 patients (42.32 ± 26.64 years old) with various non-acute CNSDs in which the effect of LMC was assessed to restore FMD in the most affected UE (2 independent comparisons [77,78]), on the least affected side (2 independent comparisons [77,78]) and bilaterally (3 independent comparisons [76,77,78]). Very low-quality evidence of no effect of LMC was found in the most affected UE (SMD = 0.37; 95% CI = −0.57, 1.33; *p* = 0.435), in the least affected UE (SMD = 0.18; 95% CI = −0.77, 1.12; *p* = 0.709), and bilateral UE (SMD = 0.01; 95% CI = −0.76, 0.77; *p* = 0.99) on FMD in comparison with CT (Table 4, Figure 5). The publication bias risk was not able to be assessed in the most and least affected UEs, and in bilateral UE, the risk was not present (Figure S4 in the online supplementary files). Heterogeneity was not found in any comparison, and the precision level was low in all meta-analyses due to the low number of participants per study.

When subgroup analysis was performed, only very low-quality evidence of a high effect of LMC and more CT (SMD = 0.82; 95% CI = 0.07, 1.57; *p* = 0.032) was found in the most affected UE and in bilateral UE (SMD = 0.91; 95% CI = 0.15, 1.66; *p* = 0.018), both versus CT, on FMD in patients with non-acute CNSD.

## 4. Discussion

Severe motor impairments in the UE, mainly muscle weakness and spasticity [81], are among the most disabling consequences that appear in patients diagnosed with CNSD and can reduce their and their families’ quality of life [82,83]. The recovery of the UE motor function of these patients is essential to improve the functional capacity and increase personal autonomy to perform ADLs. VR implementations have made possible the development of devices with therapeutic purposes, such as LMC. Only a few studies [76,77,78,79,80] have been carried out to evaluate the ability of LMC to recover UE motor function in patients with CNSD, and a meta-analysis to analyze its effect has not been performed. Therefore, we present the first systematic review with meta-analysis to assess the effect of LMC on different aspects of UE motor function in patients with CNSD, such as stroke, CP, Parkinson’s disease, or multiple sclerosis. In addition, this review provides a higher level of evidence than an isolated RCT on the use of LMC in UE rehabilitation in CNSD subjects, favoring the inclusion of LMC-based video games in novel protocols of UE motor function rehabilitation of patients with CNSDs.

LMC is postulated to be a ludic tool that could favor the inclusion of participants in a playful and different environment in which they can face new challenges and reach new goals interacting in real time with hand and finger movements [84]. It works as an active therapy that requires high commitment and motivation. LMC is a VR device that may act on brain plasticity, improving UE motor function. The brain is a complex neural network with the ability to reprogram itself through repetitive tasks [85]. Recovery of lost functions after stroke is a complex process through which the brain can reorganize itself and create new synapses between undamaged neurons [86] to restore, replace, and/or compensate for impaired functions [87]. For these reasons, it is essential to develop new effective and safe therapeutic strategies.

Initially, we assessed the effect of video game-based LMC therapy on UE mobility in patients diagnosed with stroke. Stroke is the second leading cause of death and is mainly responsible for causing large amounts of disability in adults and middle-aged people worldwide [88]. Motor impairments in the UE, such as spasticity of hemiplegia, are the main cause of disability after stroke [89] and appear in 80% of stroke patients [90], resulting in decreased functional capacity that persists at 6 months after stroke in 30–66% of stroke survivors [91]. Finding effective therapies to reduce UE motor function restrictions is crucial. In recent years, non-immersive VR tools have started to be applied in the motor neurorehabilitation of stroke patients [92]. Our results suggested that LMC could be effective on UE mobility in patients with stroke. Specifically, we found a higher effect of LMC when it was combined with CT compared to CT to improve UE mobility and UE mobility-oriented tasks. LMC requires continuous interaction between the patient and the game through UE movements, especially in the elbow, hand, and fingers. The repetitive tasks of the different games could favor brain plasticity. Different studies have suggested that non-immersive VR therapy produces improvements in UE motor function in patients with stroke and increases gray matter volume in the motor and premotor regions of the affected hemisphere and could be correlated with an improvement in motor skills in undamaged brain areas, suggesting plasticity changes related to imagining, planning, and performing motor tasks [93]. In recent studies, other non-immersive VR devices, such as Doctor Kinetic [94] and the Nintendo^®^ Wii gaming system [95], have proven effective in improving UE motor function in stroke patients, demonstrating that they are a good tool to be used in neurorehabilitation, although they are not superior to CT when used alone [96,97]. One of the main strengths of the LMC in retraining mobility in stroke patients is that it allows the recognition and reproduction of the movements of the elbow, wrist, hand, and fingers in the three planes of space with high accuracy [36], improving the overall mobility of any joint of the damaged UE. Another interest is that LMC has shown a high precision in gesture recognition in LMC-developed games, allowing training of the functional movement of damaged UE with games that include ADLs such as dressing, cooking, eating, playing the piano, garden work, and other functional activities [98]. Finally, another of the main clinical implications of the findings of this meta-analysis in patients with stroke is that when LMC is combined with CT, the effect on UE mobility oriented to performing an activity is higher compared to CT. This supports the inclusion of LMC in physiotherapy and occupational therapy protocols and the subsequent development of new LMC-based video games to be used in neurorehabilitation.

The effectiveness of LMC in the treatment of non-acute CNSD (multiple sclerosis, Parkinson’s disease, and cerebral palsy) was analyzed in other meta-analyses. Our overall findings showed a moderate effect of LMC therapy on GS in the most affected UE. However, LMC did not report an effect on GS in the least affected UE. With very low-quality evidence, we suggest that LMC may be a valid tool to train GS in subjects with CP, multiple sclerosis, or Parkinson’s disease. Quantitatively, LMC has been demonstrated to be a valid tool to improve GS in healthy subjects [99], and this meta-analysis summarizes the available evidence to postulate LMC as a non-immersive VR haptic device that is portable and inexpensive for use in physiotherapy or occupational therapy sessions. In addition, the LMC does not require the placement of sensors in the UE and it does not request manual contact interaction for use favors the movement of the damaged UE, restricted by muscle weakness or spasticity, in patients with poor or no GS and without having to overcome any resistance of the material used [33].

When LMC was used to improve the GMD in these patients with respect to CT, our findings showed its positive effects in the most affected UE, which were higher when it was combined with CT. To date, only Clutterbuck et al. [100], in a systematic review without meta-analysis, has found that non-immersive VR, such as Nintendo^®^ Wii or Sony Eye, produces a positive and weak effect for improving gross motor function in patients with cerebral palsy. Jonsdottir et al. [101], in a recent randomized controlled pilot study, reported that virtual reality in a serious gaming (Rehab@Home) approach was feasible and beneficial to the arm function of persons with multiple sclerosis. However, Chiu et al. [53] support that non-immersive VR through Nintendo^®^ Wii does not improve GS or hand function in cerebral palsy patients. The main difference between LMC and the major non-immersive VR devices, such as Nintendo^®^ Wii, is that LMC does not need a manual controller, allowing the reproduction of natural hand gestures when the patients interact with the game. This may explain the differences in gross motor recovery and GS between the use of LMC therapy or Nintendo^®^ Wii. Our results can complement the previous findings, support a guarantee to use LMC to improve GMD in subjects with cerebral palsy and other non-acute CNSDs and suggest the use of LMC with one of the best non-immersive VR devices, without manual contact, to be used in the neurorehabilitation of UE in patients with CNSDs. Unlike other VR devices, such as the Kinect^®^, which are more geared towards reproducing global movements while standing to maintain balance [39], LMC is a highly accurate and validated device to primarily rehabilitate the damaged UE [41,42].

Finally, in relation to the FMD, in an overall analysis, LMC did not produce an effect on this outcome. However, when LMC was used in combination with CT, very low-quality evidence of a higher effect in the most affected and bilateral UE was shown. Our results are consistent with those recently published by Şahin S. et al. (2020) [102], who showed that a program of non-immersive VR with more CT and occupational therapy in this case produced higher gross and FMD improvements in comparison with the use of CT alone. A previous systematic review conducted by Rathinam C. et al. (2019) [103] reported that the improvement in hand function, especially in FMD, of patients with non-acute CNSD, such as cerebral palsy, using non-immersive VR devices is weak, although it suggests that non-immersive VR devices may be used as an adjunct to CT. Currently, different haptic VR devices are being developed to train FMD in patients with non-acute CNSD. The majority of these systems use glove orthoses and button devices to press or screens to touch [104,105]; however, LMC does not need gloves or devices to be pressed or touched with the hands and fingers, allowing free fine and gross movements of their hands and fingers without mechanical restrictions. The high accuracy in the hand and finger detection position and movement [106] indicates that the LMC was integrated into very specific treatment protocols for UE rehabilitation, such as those carried out from occupational therapy.

The results derived from this review are consistent with previously published literature because, in most cases, a higher recovery in UE motor function was obtained when LMC was used in combination with CT in different CNSD patients [97,102,107]. In a recent meta-analysis, Johansen et al. (2020) [108] showed that the use of non-immersive games, including in a CT program, produced higher improvement in the disabled hand of patients with CNSD, such as cerebral palsy. LMC is also a motion-controlled video game, and combined with CT, LMC may be presented as an excellent adjuvant tool to improve UE motor function, especially manual skills, as it is able to customize and adapt the therapy to each patient, making it more specific and attractive for increasing UE recovery.

Vanbellingen et al. [32] described 4 motives for why LMC is useable. First, LMC is a lightweight, small, USB-powered device that can be plugged into every computer. Second, the installed software is easy to use. Third, no skilled technician is needed, as there is no need to place device markers on the hands, making the tool easier to use than other upper-extremity VR tools, such as exoskeletons or virtual gloves. Fourth, the LMC system is relatively inexpensive and easy to buy and can be easily integrated into the home environment, resulting in high feasibility of video game-based training with an LMC device. Due to all these advantages, in addition to our results, it would be interesting for LMC to be used in future studies in which non-immersive virtual reality treatment will be planned.

Finally, our review supports LMC as the leading haptic VR sensor for UE mobility recovery compared to other non-immersive VR devices, such as Doctor Kinetic^®^ and Nintendo^®^ Wii gaming systems, which are more specialized for posture and balance. The LMC is a highly accurate sensor; therefore, it will allow training of fine and precise hand and finger movements. The low cost of LMC favors its inclusion in well-designed home treatments and could increase the level of UE recovery in patients with difficulties accessing the rehabilitation center. In addition, LMC is being implemented to develop a 3D point of intent determination method using multimodal fusion between hand pointing and gaze for a virtual 3D display, which will expand its use to new challenges in the future [109].

Some limitations must be considered in this review. First, the low number of studies included can reduce the generalization of our findings. Second, the low sample size reduces the precision level of our findings and increases the possibility of selection bias. However, a lower sample size is a characteristic of the majority of studies published with CNSD patients. The low number of comparisons in each meta-analysis considerably decreased the quality evidence of our findings. It is important to take into account the impossibility of blinding the participants in each group, which can increase the risk of performance bias. Finally, another limitation is the difficulty of studying the risk of publication bias in meta-analyses of fewer than three studies. For further research, it will be necessary to increase the sample size in each study and perform more RCTs with the aim of obtaining more robust findings regarding the use of LMC as a successful therapy on different aspects of UE motor function, such as UE mobility, UE mobility-oriented tasks, GS, GMD, and FMD, in patients with different CNSDs. In the future, it will be essential to develop RCTs to assess hand skills in disabled UE patients with stroke.

## 5. Conclusions

Our results suggest that LMC may be considered a useful and effective a haptic VR device to improve different aspects of UE motor function in patients with CNSD. Very low-quality evidence of a large effect favoring LMC therapy compared to CT was found on UE mobility and UE mobility-oriented tasks in patients with stroke, and LMC showed large effects on these outcomes when it was used combined with CT. In patients with non-acute CNSD, our findings showed (1) low-quality evidence of a moderate effect of LMC on GS of the most affected UE in comparison with CT; (2) low-quality evidence of a moderate effect of LMC on GMD on the most affected UE, which was higher when LMC was used combined with CT in comparison with CT alone; and (3) very low-quality evidence of a large effect of LMC in combination with CT was shown on FMD of the most affected UE and bilateral CNSD. Finally, our findings postulate the higher effect of LMC on UE motor function when it is added to a CT program in patients with CNSD.

## Figures and Tables

**Table 1:** Bibliographic search strategy used in each database.

DATABASE	SEARCH STRATEGY
PubMed Medline	(“leap motion”[tiab] OR “leap motion controller”[tiab] OR leap motion sensor[tiab] OR LMC[tiab]) AND (upper extremity[mh] OR upper extremity[tiab] OR upper limb[tiab])
Web of Science	(*leap motion controller*) AND (*upper extremity* OR *upper limb*)
Scopus	[TITLE-ABS-KEY (“leap motion controller”) AND (“upper limb” OR “upper extremity”)]
PEDro	Leap motion AND upper limb
CINAHL	(AB leap motion OR AB leap motion controller) AND (AB upper extremity OR AB upper limb)

**Table 2:** Main characteristics of the studies included in the review.

					EXPERIMENTAL GROUP								COMPARISON GROUP							OUTCOMES		
					Sample				Intervention				Sample			Control						
Author and Year	Country	K	N	ND	Evol (years)	N_e_	Age	M:F	Type	Weeks	Ses/ Week	Min	N_c_	Age	M:F	Type	Weeks	Ses/ Week	Min	Type	Test	Time
Avcil, E et al. (2020) [76]	Turkey	4	30	Cerebral Palsy	NR	15	10.93	8:7	LMC + NWT	8	3	60	15	11.07	9:6	CT	8	3	60	GS	Dynamometer	Immediate
																				GMD	MMDT	
																				FMD	DHI	
Cuesta-Gómez, A. et al. (2020) [77]	Spain	7	30	Multiple Sclerosis	15.20	16	49.86	7:9	LMC + CT	10	2	60	14	42.66	5:9	CT	10	2	60	GS	Dynamometer	Immediate
																				GMD	BBT	
																				FMD	PPT	
Fernández-González, P. et al. (2019) [78]	Spain	7	23	Parkinson Disease	NR	12	65.77	6:6	LMC	6	2	30	11	73.63	5:6	CT	6	2	30	GP	Dynamometer	Immediate
																				GMD	BBT	
																				FMD	PPT	
Wang, Z. et al. (2017) [79]	China	2	26	Stroke	0.13	13	55.3	11:2	LMC + OT	4	5	45	13	53.4	11:2	CT	4	5	45	UE motor function	FM-UE	Immediate
																					ARAT	
Ögün, M.N. et al. (2019) [80]	Turkey	2	65	Stroke	0.28	33	61.48	28:5	LMC	6	3	60	32	59.75	23:9	CT +Pas VR	6	3	60	UE motor function	WMFT	Immediate

Abbreviations: K, number of comparisons; N, total sample size; ND, neurological diseases; Evol, evolution; Ne, experimental group sample size; M, male; F, female; Ses, sessions; Min, minutes; Nc, control group sample size; NR, not reported; LMC, Leap Motion Controller; NWT, Nintendo^®^ Wii Therapy; CT, conventional therapy; OT, occupational therapy; GS, grip strength; GMD, gross motor dexterity; FMD, fine motor dexterity; MMDT, Minnesota Manual Dexterity Test; DHI, Duruoz Hand Index; BBT, Box and Block Test; PPT, Purdue Pegboard Test; FM-UE, Fugl–Meyer Upper Extremity; ARAT, Action Research Arm Test; WMFT Wolf Motor Function Test; Pas VR, Passive virtual reality visualization.

**Table 3:** Cochrane Risk of Bias assessment of the included studies.

	Selection Bias		Performance Bias	Detection Bias	Attrition Bias	Reporting Bias	Other Bias
Author and Year	Random Sequence Generation	Concealment of Randomization Sequence	Blinding of Participants	Blinding of Outcomes Assessors	Incomplete Outcome Data	Selective Reporting	Other, Ideally Prespecified
Avcil, E. et al. (2020) [76]	-	+	+	?	-	-	-
Cuesta-Gómez, A. et al. (2020) [77]	-	+	+	-	-	-	-
Fernández-González, P. et al. (2019) [78]	-	+	+	+	-	-	-
Wang, Z. et al. (2017) [79]	-	+	+	-	-	-	-
Ögün, M.N. et al. (2019) [80]	-	+	+	-	-	-	-

Abbreviations: “+” = high risk of bias, “-” = low risk of bias, “?” = inadequate data for the evaluation.

**Table 4:** Main findings of each meta-analysis.

	Summary of Findings									Quality of Evidence (GRADE)					
	Pooled Effect Het						Publication Bias								
	K	N	N_s_	SMD	95% CI	*I*^2^ (*p*-Value)	(Egger *p*-Value)	Trim and Fill		Risk of Bias	Incons	Indirect	Imprec	Pub. Bias	Quality
								Adj SMD	% of Var						
**STROKE**															
**Overall UE Mobility**	2	91	45.5	0.96	0.47; 1.45	0% (0.31)	-	-	-	Medium	No	No	Yes	Likely	Very low
**Overall UE** **Oriented-Task Mobility**	2	91	45.5	1.29	0.84; 1.74	0% (0.94)	-	-	-	Medium	No	No	Yes	Likely	Very low
**NON-ACUTE CNSD (CP, MS, and PD)**															
**GRIP STRENGTH**															
**Overall Most Affected UE**	3	83	27.6	0.47	0.03; 0.90	0% (0.45)	0.49	0.47	0%	Medium	No	No	Yes	Low	Low
**Overall Least Affected UE**	3	83	27.6	0.30	−0.12; 0.74	0% (0.46)	0.58	0.30	0%	Medium	No	No	Yes	Low	Low
**GROSS MOTOR DEXTERITY**															
**Overall Most Affected UE**	3	83	27.6	0.73	0.28; 1.17	0% (0.57)	0.24	0.73	0%	Medium	No	No	Yes	Low	Low
**Overall Least Affected UE**	2	53	26.5	0.24	−0.29; 0.78	0% (0.92)	-	-	-	Medium	No	No	Yes	Likely	Very low
**FINE MOTOR DEXTERITY**															
**Overall Most Affected UE**	2	53	26.5	0.37	−0.57; 1.33	0% (0.31)	-	-	-	Medium	No	No	Yes	Likely	Very low
**Overall Least Affected UE**	2	53	26.5	0.18	−0.77; 1.12	0% (0.39)	-	-	-	Medium	No	No	Yes	Likely	Very low
**Overall Bilateral UE**	3	83	27.6	0.01	−0.76; 0.77	0% (0.38)	0.95	<0.01	0%	Medium	No	No	Yes	Low	Low

Abbreviations: GRADE, Grading of Recommendations Assessment Development and Evaluation; Het, heterogeneity; K, number of studies; N, number of participants in each meta-analysis; N_s_, mean of participants per study; SMD, Cohen standardized mean difference; CI, confidence interval; I^2^, Higgins degree of inconsistency; Adj, adjusted; Incons, inconsistency; Indirect, indirectness; Imprec, imprecision; Pub. Bias, publication bias; Sym, symmetric; Asym, asymmetric; CP, cerebral palsy; MS, multiple sclerosis; PD, Parkinson’s disease.

## Data Availability

Not applicable.

## Supplementary Materials

The following are available online at https://www.mdpi.com/1424-8220/21/6/2065/s1: Figure S1. Funnel plot of the effect of LMC-based therapy on recovery of grip strength in the most affected UE in patients with non-acute CNSD; Figure S2. Funnel plot of the effect of LMC-based therapy on recovery of grip strength in the least affected UE patients with non-acute CNSD; Figure S3. Funnel plot of the effect of LMC-based therapy on the recovery of gross motor dexterity in the most affected UE in patients with non-acute CNSD; Figure S4. Funnel plot of the effect of LMC-based therapy on the recovery of fine motor dexterity in the bilateral side in patients with non-acute CNSD.

## References

[1] Del Pozo-Cruz B., Adsuar J.C., Parraca J.A., del Pozo-Cruz J., Olivares P.R., Gusi N. (2012). Using Whole-Body Vibration Training in Patients Affected with Common Neurological Diseases: A Systematic Literature Review. J. Altern. Complement. Med..

[2] Murphy S.J., Werring D.J. (2020). Stroke: Causes and clinical features. Medicine.

[3] Rosenbaum P., Paneth N., Leviton A., Goldstein M., Bax M. (2007). A report: The definition and classification of cerebral palsy April 2006. Dev. Med. Child Neurol..

[4] Gautam R., Sharma M. (2020). Prevalence and Diagnosis of Neurological Disorders Using Different Deep Learning Techniques: A Meta-Analysis. J. Med. Syst..

[5] Baek K., Doñamayor N., Morris L.S., Strelchuk D., Mitchell S., Mikheenko Y., Yeoh S.Y., Phillips W., Zandi M., Jenaway A. (2017). Impaired awareness of motor intention in functional neurological disorder: Implications for voluntary and functional movement. Psychol. Med..

[6] Nonnekes J., Goselink R.J.M., Růžička E., Fasano A., Nutt J.G., Bloem B.R. (2018). Neurological disorders of gait, balance and posture: A sign-based approach. Nat. Rev. Neurol..

[7] Zhu W., Jiang Y. (2019). Determinants of quality of life in patients with hemorrhagic stroke: A path analysis. Medicine.

[8] Schallert W., Fluet M.-C., Kesselring J., Kool J. (2020). Evaluation of upper limb function with digitizing tablet-based tests: Reliability and discriminative validity in healthy persons and patients with neurological disorders. Disabil. Rehabil..

[9] Bakers J.N.E., van den Berg L.H., Ajeks T.G., Holleman M.J., Verhoeven J., Beelen A., Visser-Meily J.M.A., van Eijk R.P.A. (2020). Portable fixed dynamometry: Towards remote muscle strength measurements in patients with motor neuron disease. J. Neurol..

[10] Bohannon R. (2007). Muscle strength and muscle training after stroke. J. Rehabil. Med..

[11] Auld M.L., Boyd R.N., Moseley G.L., Ware R.S., Johnston L.M. (2012). Impact of Tactile Dysfunction on Upper-Limb Motor Performance in Children with Unilateral Cerebral Palsy. Arch. Phys. Med. Rehabil..

[12] Carlsson H., Gard G., Brogårdh C. (2018). Upper-limb sensory impairments after stroke: Self-reported experiences of daily life and rehabilitation. J. Rehabil. Med..

[13] Ballantyne R., Rea P.M. (2019). A Game Changer: The Use of Digital Technologies in the Management of Upper Limb Rehabilitation.

[14] Pomeroy V., Aglioti S.M., Mark V.W., McFarland D., Stinear C., Wolf S.L., Corbetta M., Fitzpatrick S.M. (2011). Neurological Principles and Rehabilitation of Action Disorders. Neurorehabil. Neural Repair.

[15] Levac D., Glegg S., Colquhoun H., Miller P., Noubary F. (2017). Virtual Reality and Active Videogame-Based Practice, Learning Needs, and Preferences: A Cross-Canada Survey of Physical Therapists and Occupational Therapists. Games Health J..

[16] Turolla A., Venneri A., Farina D., Cagnin A., Cheung V.C.K. (2018). Rehabilitation Induced Neural Plasticity after Acquired Brain Injury. Neural Plast..

[17] da Silva Ribeiro N.M., Ferraz D.D., Pedreira É., Pinheiro Í., da Silva Pinto A.C., Neto M.G., dos Santos L.R.A., Pozzato M.G.G., Pinho R.S., Masruha M.R. (2015). Virtual rehabilitation via Nintendo Wii® and conventional physical therapy effectively treat post-stroke hemiparetic patients. Top. Stroke Rehabil..

[18] Barreca S., Wolf S.L., Fasoli S., Bohannon R. (2003). Treatment Interventions for the Paretic Upper Limb of Stroke Survivors: A Critical Review. Neurorehabil. Neural Repair.

[19] Jakob I., Kollreider A., Germanotta M., Benetti F., Cruciani A., Padua L., Aprile I. (2018). Robotic and Sensor Technology for Upper Limb Rehabilitation. PM R.

[20] Levin M.F., Weiss P.L., Keshner E.A. (2015). Emergence of Virtual Reality as a Tool for Upper Limb Rehabilitation: Incorporation of Motor Control and Motor Learning Principles. Phys. Ther..

[21] Massetti T., da Silva T.D., Crocetta T.B., Guarnieri R., de Freitas B.L., Bianchi Lopes P., Watson S., Tonks J., de Mello Monteiro C.B. (2018). The Clinical Utility of Virtual Reality in Neurorehabilitation: A Systematic Review. J. Cent. Nerv. Syst. Dis..

[22] Ventura S., Brivio E., Riva G., Baños R.M. (2019). Immersive Versus Non-immersive Experience: Exploring the Feasibility of Memory Assessment through 360° Technology. Front. Psychol..

[23] Gatica-Rojas V., Méndez-Rebolledo G. (2014). Virtual reality interface devices in the reorganization of neural networks in the brain of patients with neurological diseases. Neural Regen. Res..

[24] Kim W.-S., Cho S., Park S.H., Lee J.-Y., Kwon S., Paik N.-J. (2018). A low cost kinect-based virtual rehabilitation system for inpatient rehabilitation of the upper limb in patients with subacute stroke. Medicine.

[25] Turolla A., Dam M., Ventura L., Tonin P., Agostini M., Zucconi C., Kiper P., Cagnin A., Piron L. (2013). Virtual reality for the rehabilitation of the upper limb motor function after stroke: A prospective controlled trial. J. Neuroeng. Rehabil..

[26] Kim W.-S., Cho S., Ku J., Kim Y., Lee K., Hwang H.-J., Paik N.-J. (2020). Clinical Application of Virtual Reality for Upper Limb Motor Rehabilitation in Stroke: Review of Technologies and Clinical Evidence. J. Clin. Med..

[27] Miclaus R., Roman N., Caloian S., Mitoiu B., Suciu O., Onofrei R.R., Pavel E., Neculau A. (2020). Non-Immersive Virtual Reality for Post-Stroke Upper Extremity Rehabilitation: A Small Cohort Randomized Trial. Brain Sci..

[28] Wu J., Loprinzi P.D., Ren Z. (2019). The Rehabilitative Effects of Virtual Reality Games on Balance Performance among Children with Cerebral Palsy: A Meta-Analysis of Randomized Controlled Trials. Int. J. Environ. Res. Public Health.

[29] Norouzi E., Gerber M., Pühse U., Vaezmosavi M., Brand S. (2020). Combined virtual reality and physical training improved the bimanual coordination of women with multiple sclerosis. Neuropsychol. Rehabil..

[30] Santos P., Machado T., Santos L., Ribeiro N., Melo A. (2019). Efficacy of the Nintendo Wii combination with Conventional Exercises in the rehabilitation of individuals with Parkinson’s disease: A randomized clinical trial. NeuroRehabilitation.

[31] Leemhuis E., Esposito R.M., De Gennaro L., Pazzaglia M. (2021). Go Virtual to Get Real: Virtual Reality as a Resource for Spinal Cord Treatment. Int. J. Environ. Res. Public Health.

[32] Vanbellingen T., Filius S.J., Nyffeler T., van Wegen E.E.H. (2017). Usability of Videogame-Based Dexterity Training in the Early Rehabilitation Phase of Stroke Patients: A Pilot Study. Front. Neurol..

[33] Khademi M., Mousavi Hondori H., McKenzie A., Dodakian L., Lopes C.V., Cramer S.C. (2014). Free-hand interaction with leap motion controller for stroke rehabilitation. Proceedings of the CHI ’14 Extended Abstracts on Human Factors in Computing Systems.

[34] Niechwiej-Szwedo E., Gonzalez D., Nouredanesh M., Tung J. (2018). Evaluation of the Leap Motion Controller during the performance of visually-guided upper limb movements. PLoS ONE.

[35] Oña E.D., Balaguer C., Cano-de la Cuerda R., Collado-Vázquez S., Jardón A. (2018). Effectiveness of Serious Games for Leap Motion on the Functionality of the Upper Limb in Parkinson’s Disease: A Feasibility Study. Comput. Intell. Neurosci..

[36] Bachmann D., Weichert F., Rinkenauer G. (2018). Review of Three-Dimensional Human-Computer Interaction with Focus on the Leap Motion Controller. Sensors.

[37] Jiang X., Xu W., Sweeney L., Li Y., Gross R., Yurovsky D. New directions in contact free hand recognition. Proceedings of the 2007 IEEE International Conference on Image Processing.

[38] Han J., Gold N.E. (2014). Lessons Learned in Exploring the Leap MotionTM Sensor for Gesture-Based Instrument Design.

[39] Skals S., Rasmussen K.P., Bendtsen K.M., Yang J., Andersen M.S. (2017). A musculoskeletal model driven by dual Microsoft Kinect Sensor data. Multibody Syst. Dyn..

[40] Bachmann D., Weichert F., Rinkenauer G. (2014). Evaluation of the leap motion controller as a new contact-free pointing device. Sensors.

[41] Cikajlo I., Peterlin Potisk K. (2019). Advantages of using 3D virtual reality based training in persons with Parkinson’s disease: A parallel study. J. Neuroeng. Rehabil..

[42] Kim S., Park S., Lee O. (2020). Development of a Diagnosis and Evaluation System for Hemiplegic Patients Post-Stroke Based on Motion Recognition Tracking and Analysis of Wrist Joint Kinematics. Sensors.

[43] Ferreira S.C., Chaves R.O., Seruffo M.C.d.R., Pereira A., Azar A.P.D.S., Dias Â.V., Dos Santos A.D.A.S., Brito M.V.H. (2020). Empirical Evaluation of a 3D Virtual Simulator of Hysteroscopy Using Leap Motion for Gestural Interfacing. J. Med. Syst..

[44] Nizamis K., Rijken N., Mendes A., Janssen M., Bergsma A., Koopman B. (2018). A Novel Setup and Protocol to Measure the Range of Motion of the Wrist and the Hand. Sensors.

[45] Weichert F., Bachmann D., Rudak B., Fisseler D. (2013). Analysis of the Accuracy and Robustness of the Leap Motion Controller. Sensors.

[46] Smeragliuolo A.H., Hill N.J., Disla L., Putrino D. (2016). Validation of the Leap Motion Controller using markered motion capture technology. J. Biomech..

[47] Chophuk P., Chumpen S., Tungjitkusolmun S., Phasukkit P. Hand postures for evaluating trigger finger using leap motion controller. Proceedings of the BMEiCON 2015—8th Biomedical Engineering International Conference.

[48] Fonk R., Schneeweiss S., Simon U., Engelhardt L. (2021). Hand Motion Capture from a 3D Leap Motion Controller for a Musculoskeletal Dynamic Simulation. Sensors.

[49] Gamboa E., Serrato A., Castro J., Toro D., Trujillo M. (2020). Advantages and Limitations of Leap Motion from a Developers’, Physical Therapists’, and Patients’ Perspective. Methods Inf. Med..

[50] Iosa M., Morone G., Fusco A., Castagnoli M., Fusco F.R., Pratesi L., Paolucci S. (2015). Leap motion controlled videogame-based therapy for rehabilitation of elderly patients with subacute stroke: A feasibility pilot study. Top. Stroke Rehabil..

[51] Johnson L., Bird M.-L., Muthalib M., Teo W.-P. (2020). An Innovative STRoke Interactive Virtual thErapy (STRIVE) Online Platform for Community-Dwelling Stroke Survivors: A Randomized Controlled Trial. Arch. Phys. Med. Rehabil..

[52] Schuster-Amft C., Eng K., Suica Z., Thaler I., Signer S., Lehmann I., Schmid L., McCaskey M.A., Hawkins M., Verra M.L. (2018). Effect of a four-week virtual reality-based training versus conventional therapy on upper limb motor function after stroke: A multicenter parallel group randomized trial. PLoS ONE.

[53] Chiu H.-C., Ada L., Lee H.-M. (2014). Upper limb training using Wii Sports Resort ^TM^ for children with hemiplegic cerebral palsy: A randomized, single-blind trial. Clin. Rehabil..

[54] Karamians R., Proffitt R., Kline D., Gauthier L.V. (2020). Effectiveness of Virtual Reality- and Gaming-Based Interventions for Upper Extremity Rehabilitation Poststroke: A Meta-analysis. Arch. Phys. Med. Rehabil..

[55] Domínguez-Téllez P., Moral-Muñoz J.A., Salazar A., Casado-Fernández E., Lucena-Antón D. (2020). Game-Based Virtual Reality Interventions to Improve Upper Limb Motor Function and Quality of Life After Stroke: Systematic Review and Meta-analysis. Games Health J..

[56] Webster A., Poyade M., Rooney S., Paul L. (2021). Upper limb rehabilitation interventions using virtual reality for people with multiple sclerosis: A systematic review. Mult. Scler. Relat. Disord..

[57] Tarakci E., Arman N., Tarakci D., Kasapcopur O. (2019). Leap Motion Controller–based training for upper extremity rehabilitation in children and adolescents with physical disabilities: A randomized controlled trial. J. Hand Ther..

[58] Karashanov A., Manolova A., Neshov N. (2016). Application for hand rehabilitation using Leap Motion Sensor based on a gamification approach. Int. J. Adv. Res. Sci. Eng..

[59] Moher D., Liberati A., Tetzlaff J., Altman D.G. (2009). Preferred Reporting Items for Systematic Reviews and Meta-Analyses: The PRISMA Statement. J. Clin. Epidemiol..

[60] Higgins J.P.T., Green S. (2011). Cochrane Handbook for Systematic Reviews of Intervention Version 5.1.0 [Updated March 2011].

[61] Obrero-Gaitán E., Osuna-Pérez M.C., Zagalaz-Anula N., Cortés-Pérez I., Montor-Cárdenas D. Leap Motion Controller Video Game Based Therapy for Upper Limb Rehabilitation in Patients with Neurological Disorders. A Systematic Review with Meta-analysis. PROSPERO 2020 CRD42020200771. https://www.crd.york.ac.uk/prospero/display_record.php?RecordID=200771.

[62] Hozo S.P., Djulbegovic B., Hozo I. (2005). Estimating the mean and variance from the median, range, and the size of a sample. BMC Med. Res. Methodol..

[63] Atkins D., Best D., Briss P.A., Eccles M., Falck-Ytter Y., Flottorp S., Guyatt G.H., Harbour R.T. (2004). Grading quality of evidence and strength of recommendations. BMJ.

[64] Meader N., King K., Llewellyn A., Norman G., Brown J., Rodgers M., Moe-Byrne T., Higgins J.P., Sowden A., Stewart G. (2014). A checklist designed to aid consistency and reproducibility of GRADE assessments: Development and pilot validation. Syst. Rev..

[65] Higgins J.P.T., Altman D.G., Gotzsche P.C., Juni P., Moher D., Oxman A.D., Savovic J., Schulz K.F., Weeks L., Sterne J.A.C. (2011). The Cochrane Collaboration’s tool for assessing risk of bias in randomised trials. BMJ.

[66] Higgins J.P.T., Thompson S.G., Deeks J.J., Altman D.G. (2003). Measuring inconsistency in meta-analyses. BMJ.

[67] Borenstein M., Hedges L., Higgins J., Rothstein H. Comprehensive Meta-Analysis Software Version 3. https://www.meta-analysis.com/.

[68] Cooper H., Hedges L.V., Valentine J.C. (2009). The Handbook of Research Synthesis and Meta-Analysis.

[69] DerSimonian R., Laird N. (1986). Meta-analysis in clinical trials. Control. Clin. Trials.

[70] (1977). Cohen, J Statistical Power Analysis for the Behavioral Sciences.

[71] Faraone S.V. (2008). Interpreting estimates of treatment effects: Implications for managed care. P T.

[72] Rücker G., Schwarzer G. (2020). Beyond the forest plot: The drapery plot. Res. Synth. Methods.

[73] Sterne J.A.C., Egger M. (2001). Funnel plots for detecting bias in meta-analysis: Guidelines on choice of axis. J. Clin. Epidemiol..

[74] Egger M., Smith G.D., Schneider M., Minder C. (1997). Bias in meta-analysis detected by a simple, graphical test measures of funnel plot asymmetry. BMJ.

[75] Higgins J., Thompson S., Deeks J., Altman D. (2002). Statistical heterogeneity in systematic reviews of clinical trials: A critical appraisal of guidelines and practice. J. Heal. Serv. Res. Policy.

[76] Avcil E., Tarakci D., Arman N., Tarakci E. (2020). Upper extremity rehabilitation using video games in cerebral palsy: A randomized clinical trial. Acta Neurol. Belg..

[77] Cuesta-Gómez A., Sánchez-Herrera-Baeza P., Oña-Simbaña E.D., Martínez-Medina A., Ortiz-Comino C., Balaguer-Bernaldo-de-Quirós C., Jardón-Huete A., Cano-de-la-Cuerda R. (2020). Effects of virtual reality associated with serious games for upper limb rehabilitation in patients with multiple sclerosis: Randomized controlled trial. J. Neuroeng. Rehabil..

[78] Fernández-González P., Carratalá-Tejada M., Monge-Pereira E., Collado-Vázquez S., Sánchez-Herrera Baeza P., Cuesta-Gómez A., Oña-Simbaña E.D., Jardón-Huete A., Molina-Rueda F., Balaguer-Bernaldo de Quirós C. (2019). Leap motion controlled video game-based therapy for upper limb rehabilitation in patients with Parkinson’s disease: A feasibility study. J. Neuroeng. Rehabil..

[79] Wang Z., Wang P., Xing L., Mei L., Zhao J., Zhang T. (2017). Leap Motion-based virtual reality training for improving motor functional recovery of upper limbs and neural reorganization in subacute stroke patients. Neural Regen. Res..

[80] Ögün M.N., Kurul R., Yasar M.F., Turkoglu S.A., Avci Ş., Yildiz N. (2019). Effect of Leap Motion-based 3D Immersive Virtual Reality Usage on Upper Extremity Function in Ischemic Stroke Patients. Arq. Neuropsiquiatr..

[81] Pilla A., Trigili E., McKinney Z., Fanciullacci C., Malasoma C., Posteraro F., Crea S., Vitiello N. (2020). Robotic Rehabilitation and Multimodal Instrumented Assessment of Post-stroke Elbow Motor Functions—A Randomized Controlled Trial Protocol. Front. Neurol..

[82] Manuli A., Maggio M.G., Tripoli D., Gullì M., Cannavò A., La Rosa G., Sciarrone F., Avena G., Calabrò R.S. (2020). Patients’ perspective and usability of innovation technology in a new rehabilitation pathway: An exploratory study in patients with multiple sclerosis. Mult. Scler. Relat. Disord..

[83] Bai Z., Fong K.N.K., Zhang J.J., Chan J., Ting K.H. (2020). Immediate and long-term effects of BCI-based rehabilitation of the upper extremity after stroke: A systematic review and meta-analysis. J. Neuroeng. Rehabil..

[84] Viglialoro R.M., Condino S., Turini G., Mamone V., Carbone M., Ferrari V., Ghelarducci G., Ferrari M., Gesi M. (2020). Interactive serious game for shoulder rehabilitation based on real-time hand tracking. Technol. Health Care.

[85] Gotz M., Jarriault S. (2017). Programming and reprogramming the brain: A meeting of minds in neural fate. Development.

[86] Voss P., Thomas M.E., Cisneros-Franco J.M., de Villers-Sidani E. (2017). Dynamic Brains and the Changing Rules of Neuroplasticity: Implications for Learning and Recovery. Front. Psychol..

[87] Hara Y. (2015). Brain plasticity and rehabilitation in stroke patients. J. Nippon Med. Sch..

[88] Lindmark A., Norrving B., Eriksson M. (2020). Socioeconomic status and survival after stroke—Using mediation and sensitivity analyses to assess the effect of stroke severity and unmeasured confounding. BMC Public Health.

[89] Langhorne P., Coupar F., Pollock A. (2009). Motor recovery after stroke: A systematic review. Lancet Neurol..

[90] Sommerfeld D.K., Eek E.U.-B., Svensson A.-K., Holmqvist L.W., von Arbin M.H. (2004). Spasticity After Stroke. Stroke.

[91] Bressi F., Bravi M., Campagnola B., Bruno D., Marzolla A., Santacaterina F., Miccinilli S., Sterzi S. (2020). Robotic treatment of the upper limb in chronic stroke and cerebral neuroplasticity: A systematic review. J. Biol. Regul. Homeost. Agents.

[92] Lee H.-S., Lim J.-H., Jeon B.-H., Song C.-S. (2020). Non-immersive Virtual Reality Rehabilitation Applied to a Task-oriented Approach for Stroke Patients: A Randomized Controlled Trial. Restor. Neurol. Neurosci..

[93] Keller J., Štětkářová I., Macri V., Kühn S., Pětioký J., Gualeni S., Simmons C.D., Arthanat S., Zilber P. (2020). Virtual reality-based treatment for regaining upper extremity function induces cortex grey matter changes in persons with acquired brain injury. J. Neuroeng. Rehabil..

[94] Long Y., Ouyang R., Zhang J. (2020). Effects of virtual reality training on occupational performance and self-efficacy of patients with stroke: A randomized controlled trial. J. Neuroeng. Rehabil..

[95] García Muñoz C., Casuso Holgado M.J. (2019). Efectividad de la Wii Fit Balance frente a otras intervenciones para la recuperación del equilibrio en pacientes postictus. Revisión sistemática y metaanálisis. Rev. Neurol..

[96] Saposnik G., Cohen L.G., Mamdani M., Pooyania S., Ploughman M., Cheung D., Shaw J., Hall J., Nord P., Dukelow S. (2016). Efficacy and safety of non-immersive virtual reality exercising in stroke rehabilitation (EVREST): A randomised, multicentre, single-blind, controlled trial. Lancet Neurol..

[97] Laver K.E., Lange B., George S., Deutsch J.E., Saposnik G., Crotty M. (2017). Virtual reality for stroke rehabilitation. Cochrane database Syst. Rev..

[98] Psychouli P., Katzis K., Elliott M. (2018). Home-Based Training Support for Stroke Patients Using the Leap Motion and StandInExercise Stand. Stud. Health Technol. Inform..

[99] Bostanci H., Emir A., Tarakci D., Tarakci E. (2020). Video game-based therapy for the non-dominant hand improves manual skills and grip strength. Hand Surg. Rehabil..

[100] Clutterbuck G., Auld M., Johnston L. (2019). Active exercise interventions improve gross motor function of ambulant/semi-ambulant children with cerebral palsy: A systematic review. Disabil. Rehabil..

[101] Jonsdottir J., Bertoni R., Lawo M., Montesano A., Bowman T., Gabrielli S. (2018). Serious games for arm rehabilitation of persons with multiple sclerosis. A randomized controlled pilot study. Mult. Scler. Relat. Disord..

[102] Şahin S., Köse B., Aran O.T., Bahadır Ağce Z., Kayıhan H. (2020). The Effects of Virtual Reality on Motor Functions and Daily Life Activities in Unilateral Spastic Cerebral Palsy: A Single-Blind Randomized Controlled Trial. Games Health J..

[103] Rathinam C., Mohan V., Peirson J., Skinner J., Nethaji K.S., Kuhn I. (2019). Effectiveness of virtual reality in the treatment of hand function in children with cerebral palsy: A systematic review. J. Hand Ther..

[104] Liu X., Zhu Y., Huo H., Wei P., Wang L., Sun A., Hu C., Yin X., Lv Z., Fan Y. (2019). Design of Virtual Guiding Tasks With Haptic Feedback for Assessing the Wrist Motor Function of Patients With Upper Motor Neuron Lesions. IEEE Trans. Neural Syst. Rehabil. Eng..

[105] McCall J.V., Ludovice M.C., Blaylock J.A., Kamper D.G. A Platform for Rehabilitation of Finger Individuation in Children with Hemiplegic Cerebral Palsy. Proceedings of the 2019 IEEE 16th International Conference on Rehabilitation Robotics (ICORR).

[106] Guna J., Jakus G., Pogačnik M., Tomažič S., Sodnik J. (2014). An Analysis of the Precision and Reliability of the Leap Motion Sensor and Its Suitability for Static and Dynamic Tracking. Sensors.

[107] Lee H.S., Park Y.J., Park S.W. (2019). The Effects of Virtual Reality Training on Function in Chronic Stroke Patients: A Systematic Review and Meta-Analysis. Biomed Res. Int..

[108] Johansen T., Strøm V., Simic J., Rike P. (2020). Effectiveness of training with motion-controlled commercial video games for hand and arm function in people with cerebral palsy: A systematic review and meta-analysis. J. Rehabil. Med..

[109] Yeamkuan S., Chamnongthai K. (2021). 3D Point-of-Intention Determination Using a Multimodal Fusion of Hand Pointing and Eye Gaze for a 3D Display. Sensors.

