# Supplementary material for: Leap Motion Controller Video Game-Based Therapy for Upper Extremity Motor Recovery in Patients with Central Nervous System Diseases. A Systematic Review with Meta-Analysis
Source: Sensors (Basel). 2021 Mar 15;21(6):2065. doi: 10.3390/s21062065 (PMC7999275; doi:10.3390/s21062065)
Supplement: Supplementary file 1 [file sensors-21-02065-s001.pdf]

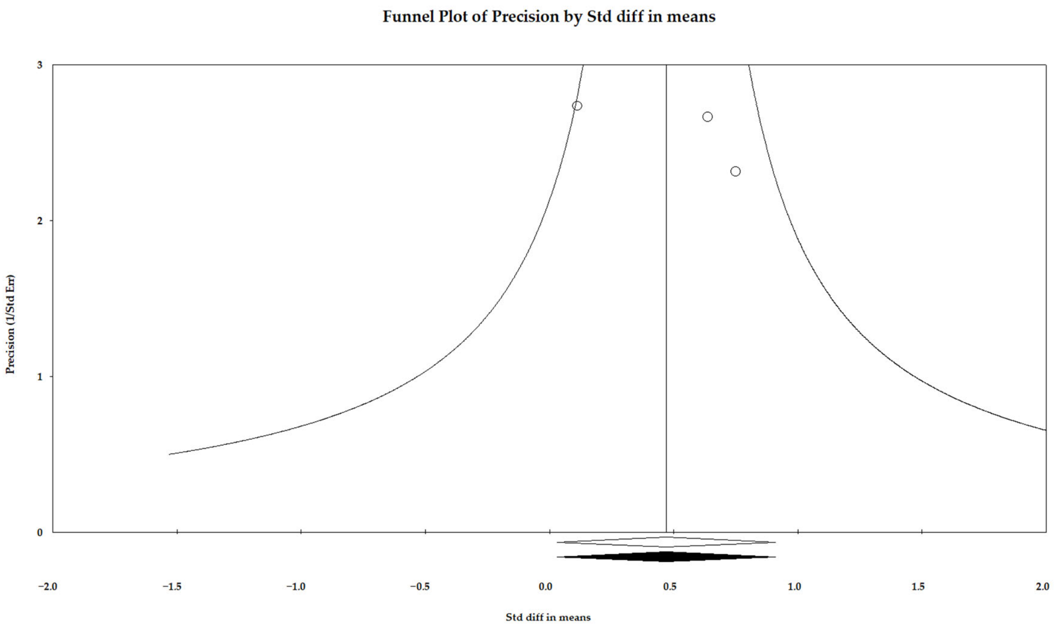

Figure S1. Funnel Plot of the Effect of LMC-based Therapy to Recover the Grip Strength in the Most Affected UE in Patients with non-acute CNSD.

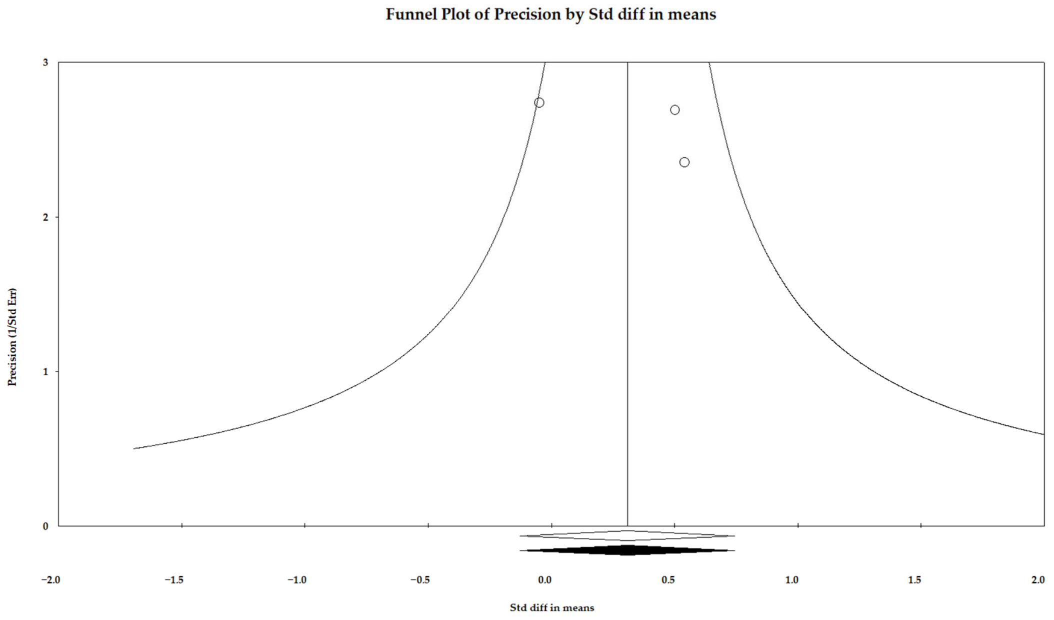

Figure S2. Funnel Plot of the Effect of LMC-based Therapy to Recover the Grip Strength in the Least Affected UE in Patients with non-acute CNSD.

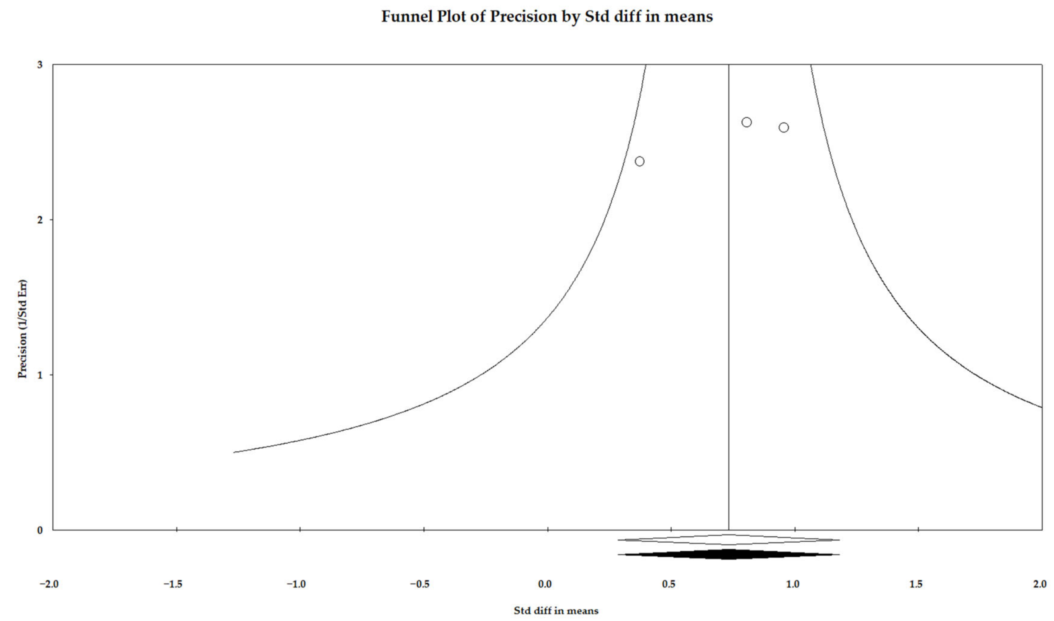

Figure S3. Funnel Plot of the Effect of LMC-based Therapy to Recover the Gross Motor Dexterity in the Most Affected UE in Patients with non-acute CNSD.

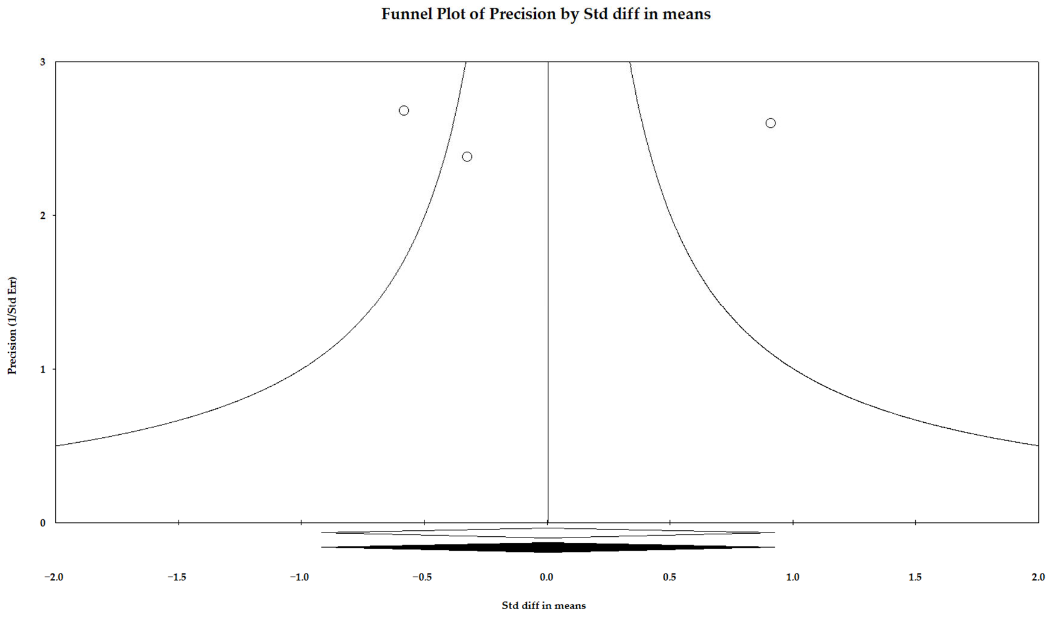

Figure S4. Funnel Plot of the Effect of LMC-based Therapy to Recover the Fine Motor Dexterity in Bilateral Side in Patients with non-acute CNSD.
